# Transient Features in Nanosecond Pulsed Electric Fields Differentially Modulate Mitochondria and Viability

**DOI:** 10.1371/journal.pone.0051349

**Published:** 2012-12-21

**Authors:** Stephen J. Beebe, Yeong-Jer Chen, Nova M. Sain, Karl H. Schoenbach, Shu Xiao

**Affiliations:** 1 Frank Reidy Research Center for Bioelectrics, Old Dominion University, Norfolk, Virginia, United States of America; 2 Department of Electrical and Computing Engineering, Old Dominion University, Norfolk, Virginia, United States of America; Cinvestav-IPN, Mexico

## Abstract

It is hypothesized that high frequency components of nanosecond pulsed electric fields (nsPEFs), determined by transient pulse features, are important for maximizing electric field interactions with intracellular structures. For monopolar square wave pulses, these transient features are determined by the rapid rise and fall of the pulsed electric fields. To determine effects on mitochondria membranes and plasma membranes, N1-S1 hepatocellular carcinoma cells were exposed to single 600 ns pulses with varying electric fields (0–80 kV/cm) and short (15 ns) or long (150 ns) rise and fall times. Plasma membrane effects were evaluated using Fluo-4 to determine calcium influx, the only measurable source of increases in intracellular calcium. Mitochondria membrane effects were evaluated using tetramethylrhodamine ethyl ester (TMRE) to determine mitochondria membrane potentials (ΔΨm). Single pulses with short rise and fall times caused electric field-dependent increases in calcium influx, dissipation of ΔΨm and cell death. Pulses with long rise and fall times exhibited electric field-dependent increases in calcium influx, but diminished effects on dissipation of ΔΨm and viability. Results indicate that high frequency components have significant differential impact on mitochondria membranes, which determines cell death, but lesser variances on plasma membranes, which allows calcium influxes, a primary determinant for dissipation of ΔΨm and cell death.

## Introduction

An hypothesis concerning effects of pulsed electric fields (PEFs) on cells states that there are increasing probabilities for electric field interactions with subcellular structures when pulse durations are in sub-microsecond ranges, shorter than the characteristic charging times for cell outer membranes [1] (typically 70–100 ns for cells in growth medium and 1 µs for tissues). This hypothesis was initially supported by showing that nsPEFs generated “sparkler cells” when intracellular granules were permeabilized in calcein loaded cells [1]. Calcein did not leak out of cells, suggesting that plasma membranes remained intact. However, modeling studies predicted that these pulses would induce “supra-electroporation” defined as high densities of very small pores in all cell membranes [2] that would lead to apoptosis [3], which had been demonstrated *in vitro* [4]–[6] and *in vivo* [7], [8]. It was also predicted that for organelles with lower membrane dielectric permittivity than that of outer membrane, such as mitochondria, the voltage induced on organelle membranes can exceed that of outer membranes [9], possibly making poration pervasive across whole cells. It was later determined that nsPEFs formed nanopores (nanometer-sized pores) in plasma membranes that were too small to allow propidium iodide or calcein to cross plasma membranes [10].

The hypothesis states that monopolar, square wave pulses with durations short compared to the charging time of outer cell membranes are implicitly characterized by rise and fall times which are short compared to the charging time. The rapidly changing electric fields during the rise and fall of the pulse amplitudes, or the transient features, are the cause for intracellular effects [1]. It was further hypothesized that the plateau of the square wave pulse determines only the magnitude of outer membrane effects, such as electropermeabilization. However, it should be pointed out that electrical pulses, which extend in duration beyond the charging time constant of cell outer membranes, can still affect subcellular structures. By porating outer membranes, an effect which is usually attributed to pulses of microsecond duration and longer, and therefore making it “transparent” for the electric field, resistive coupling with subcellular structures may cause similar effects as described above [11].

That the transient features of nanosecond pulses determine the magnitude of intracellular effects can also be understood by considering the pulses in the frequency domain. Based on the Fourier analysis of monopolar square wave pulses, assuming that they can be approximated by a trapezoidal shape with τ_p_ defining the pulse duration excluding the rising and falling part, and τ_r_ the rise and fall time, respectively, the frequency spectrum is defined by two corner frequencies. The first corner frequency, determined by the pulse duration, τ_p_, is:
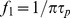



Beyond this corner frequency the spectrum decreases linearly with the frequency. For frequencies beyond the second corner frequency, which is defined by the rise and fall time, τ_r_:
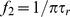
the spectrum decreases with the square of frequency. Reducing the rise and fall time therefore shifts the second corner frequency towards higher values, or in other words, adds to the high frequency components of the spectrum [12]. Reducing the rise and fall time, respectively, is therefore identical to increasing the high frequency part of the electric field pulse. Subcellular effects can be expected for frequencies exceeding the β-relaxation frequency of the plasma membrane [13]:



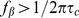
where τ_c_ is the charging time constant of the membrane. For a 600 ns pulse with a rise time of 15 ns, the second corner frequency is 21 MHz; for the same pulse duration but a rise time of 150 ns it is ten times lower at 2.1 MHz. With a β-relaxation frequency of the outer membrane of approximately of 2.1 MHz (assuming a charging time constant of the outer membrane of 75 ns) the pulse with the shorter rise and fall time has a spectrum which reaches much further beyond the 2.1 MHz, than that with the longer rise and fall time.

Since nsPEFs ablated cancer cells and tumors, it was of interest to determine more specifically whether intracellular membranes are modified due to fast transients and/or plasma membrane modifications determined by the amplitude and duration of the pulse correlated with cell death. Long lasting nanopores in plasma membranes [10], [14] or electric field-mediated plasma membrane breaching without repair could lead to cell death [15]. Damage to mitochondrial membranes could induce cell death. Dissipation of ΔΨm has been observed in response to nsPEFs [16]–[18] and cytochrome *c* was released [5], [17], but not in all cells [16]. NsPEF-induced increases in intracellular calcium have been observed through influx through plasma membranes and from endoplasmic reticulum (ER) [19]–[23]. As a second messenger calcium regulates a wide range of cell responses and can overload mitochondria, leading to cell death [24] by disrupting mitochondria calcium homeostasis. Thus, a direct or indirect path from mitochondria can lead to cell death by apoptosis and/or programmed necrosis [25], [26].

For ablation therapy, it is most likely preferable to induce both plasma membrane- and organelle-related pathways, rather than focusing on them individually. Sub-microsecond pulses with short rise and fall times (<100 ns) and longer plateaus appear to be a probable option. In pulse short-rising and short falling phase, a high probability of effects on cell interiors may be expected prior and after completion of outer membrane charging. In the plateau phase of the pulse, effects may mostly occur in outer membranes due to nanoporation and depolarization of the cell membrane potential. Multiple pathological pathways can therefore be initiated through a single or multiple pulses with a fast rise and fall waveform.

In order to explore the effect of rise and fall time on cells, we applied single 600 ns long pulses with electric field amplitudes up to 80 kV/cm. Pulses where the rise and fall times were short (15 ns) or long (150 ns) were applied to cells. The energy deposition of the 600 ns pulses were only slightly affected by changing pulse transient features. Therefore, differences observed here for cell membrane and viability effects can be considered to be energy-independent. Using a single pulse avoided electrosensitization effects that occur with multiple pulses [27]. Plasma membrane permeability changes were determined using Fluo-4 to detect calcium influx, because calcium release from intracellular stores was not observed. Effects on mitochondria membranes were determined using TMRE to determine mitochondria membrane potential (ΔΨm). Cell viability was determined in parallel experiments.

## Materials and Methods

### Cell Culture

N1-S1 hepatocellular carcinoma cells were purchased from ATCC and cultured in Iscove’s Modified Dulbecco’s Medium (ATCC, Manassas, VA) containing 10% fetal bovine serum (FBS) (Atlanta Biologist, Nocross, GA) and 300 ug/ml G-418 Sulfate (Promega, Madison, WI). Cells were permanently transfected with a luciferase plasmid (gWiz™ High Expression Reporter Plasmids, Aldevron, Fargo, ND) and identified as a clone that expressed high luciferase activity. The presence of the luciferase expression plasmid is intended for in vivo studies. The cells were maintained in a humidified incubator with 5% CO_2_ at 37°C.

### Treatment of Cells with nsPEFs

**Figure 1:**
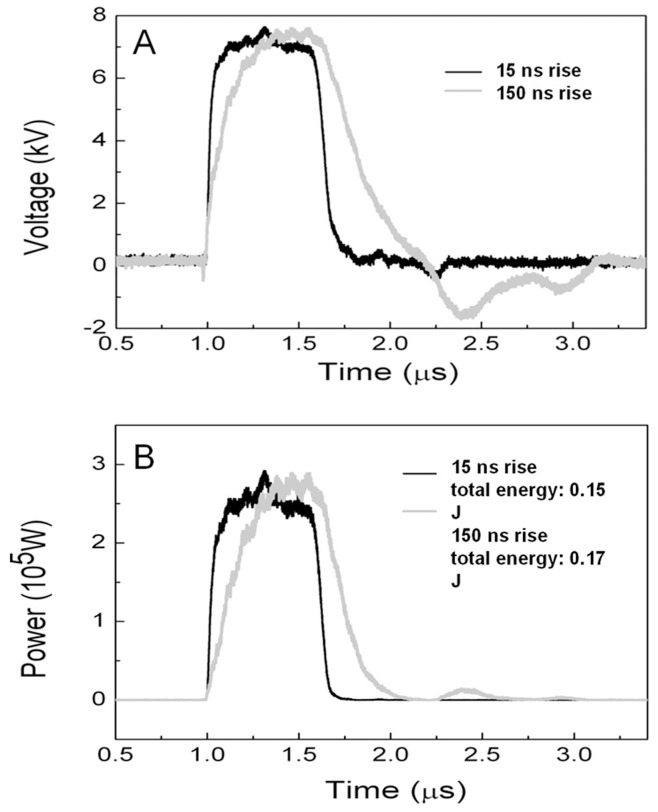
The electrical pulsing conditions used in this study. a) The 600 ns pulses with 15 ns (dark line) and 150 ns rise time (gray line). The pulses with 15 ns rise time were obtained under the best impedance-matching condition. The 150 ns rise time was obtained by adding an inductance in series with the cuvette, which caused a slight impedance mismatch and therefore a small negative tail. b) The instantaneous power of the electric pulses for the 15 ns and 150 rise times. The total energy was calculated as 0.15 J and 0.17 J for pulses with 15 ns and 150 ns rise times, respectively.

Cells were resuspended in the fresh cell culture medium at a density of 2×10^6^ cells/ml. 130 µl of cell suspension was placed in a 0.1-cm gap-width cuvette (Biosmith, San Diego, CA) and exposed to nsPEFs. N1-S1 cells were exposed or not to one pulse with duration of 600 ns and electric field strength ranging from 0 to 80 kV/cm. Pulse rise times were adjusted to either 15 ns or 150 ns rise times. A 28-stage pulse forming network (PFN) comprised of 2 nF capacitors and 128 nH stray inductors and was used to produce the 600 ns pulses to the cuvette as the load. The voltage across the cuvette is equal to half of the charging voltage under the matched-impedance condition. So the PFN was charged from 0–16 kV to produce electric fields up to 80 kV/cm across the sample in the 0.1 cm cuvettes. The impedance of the PFN is approximately 8.5 Ω, which requires the sample (cuvette) resistance to be 8.5 Ω in order to generate square pulses without tails. In actuality, the loaded cuvette has a resistance of approximately 20 Ω. This value was obtained by Ohm’s law, R = V/I. A square voltage pulse of 600 ns was applied to the cuvette and the voltage (V) and the resulting current (I) were measured. The voltage probe is Tektronix P6015 (40 kV, 75 MHz) and the current sensor is Pearson wide band current monitor (Model 5046, 20 ns). We varied the magnitude of the voltage pulse and found that the current changed linearly. From the voltage and current values in the plateau phase, the resistance was calculated as 20 Ω. To match the impedance of the PFN (8.5 Ω), a resistance of 15 Ω would need to be placed in parallel with the cuvette to reduce the load resistance to 8.5 Ω. We then varied the transient features of the pulses, i.e., the rise time and fall time. They are for a resistive load dependent on the closing speed of the switch in the PFN. In this study, a spark gap switch in atmospheric pressure air was used. We determined that the pulse rise time and falltime were approximately 15 ns (Figure 1A, dark line). As our major motivation was to study the impact of pulse transients, we deliberately added an inductance in series to the cuvette, resulting in the rise time of 150 ns (Figure1A, gray line). One consequence of this additional reactive component was the appearance of the negative tail with <20% of the peak voltage for the long (150 ns) rise time pulses. The overall energy deposition into the sample was 1.5 J and 1.7 J (Figure 1B) for the 15 ns and 150 ns transient cases, almost identical with a difference of approximately 10%. We note that for applying just single pulses, the temperature rise is estimated to be 3°C. Thus, we can rule out the role of temperature for such small increases.

### Determination of Cell Viability

N1-S1 cells were seeded at 10^5^ cells per well in 96-well plates after nsPEF treatment. Twenty four hours after treatment, cell viability was measured according to the manufacturer’s protocol by CellTiter-Glo Luminescent Cell Viability Assay Kit (Promega, Madison, WI). The luminescent signal was analyzed in luminometer (Gemini XPS, Molecular Devices, Sunnyvale, CA).

### Determination of Propidium Iodide (PI) Uptake

Cells were exposed to single nsPEFs, PI was added to a final concentration of 2.5 µg/ml immediately after pulsing and cell are analyzed by flow cytometry 10 minutes after nsPEF treatment.

### Flow Cytometry Analysis of Calcium and Mitochondrial Membrane Potential (Δψm)

The levels of intracellular calcium were determined using Fluo-4 Direct (Molecular Probes, Eugene, Oregon). Depolarization of the ΔΨm was detected using tetramethylrhodamine ethyl ester (TMRE) (Immunochemistry Technologies LLC, Bloomington, MN). Cells were preincubated with 1× Fluo-4 Direct calcium reagent loading solution for 60 min at 37°C. During the last 15 min of Fluo-4 incubation, 200 nM TMRE was added and allowed to incubate for 15 min. At the end of incubation, cells were washed and resuspended in culture media. Cells were then exposed to one 600 ns pulse with either a short or long rise time and electric fields ranging from 0–80 kV/cm. Flow cytometric analysis was performed 10 minutes after treatment with nsPEFs. The average red fluorescence intensity (10,000 cells) was analyzed on the FL-2 channel and green fluorescence was read on the FITC channel of a Becton Dickinson FacsAria flow cytometer. The green emission of Fluo-4 increases when calcium increases and TMRE red emissions decreases when ΔΨm decreases. To determine Δψm in the presence and absence of calcium, cells were preincubated with or without 5 mM EGTA for 30 minutes followed by nsPEFs treatment.

### Statistical Analysis

All experiments were performed at least two times independently in this study. All the data were expressed as mean±standard error (S.E.). Statistical differences between control and treated groups were analyzed by ANOVA with Tukey’s post-hoc correction, with p<0.05 regarded as statistically significant. For studies with multiple treatment conditions, statistical differences post-pulsing were analyzed by two-way ANOVA with Tukey’s post-hoc correction, with significance determined when p<0.05.

## Results

### EGTA Prevents Increases in Intracellular Calcium and Prevents Increases in Forward Light Scatter

**Figure 2:**
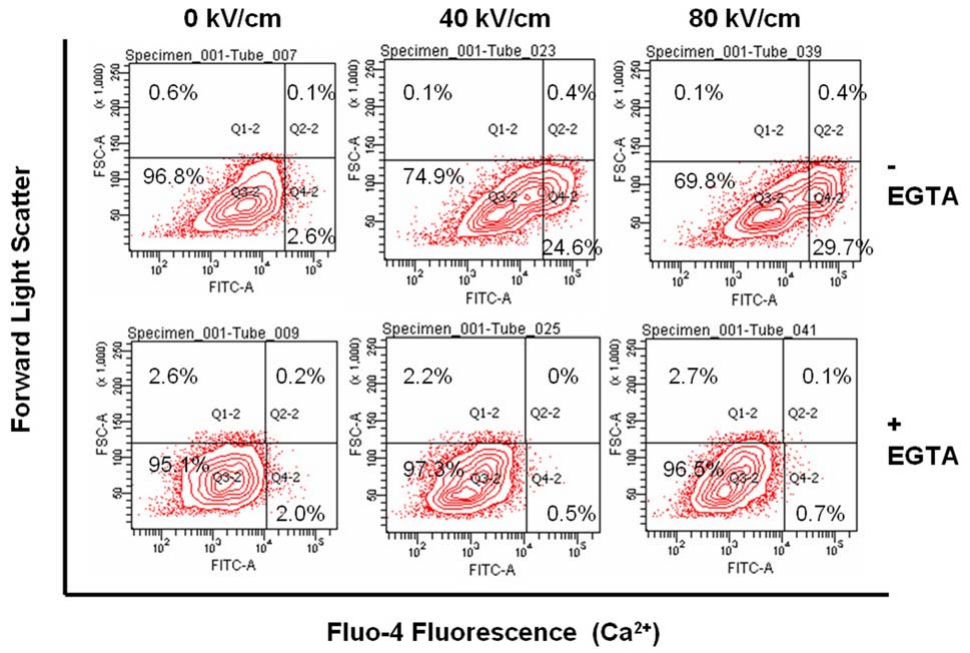
Effects of EGTA on calcium mobilization and forward light scatter in response to nsPEFs. Cells were prepared for analysis of calcium with Fluo-4 with and without EGTA as indicated in Materials and Methods EGTA. N1-S1 cells were then treated with a single 600 ns pulse at the indicated electric fields. Ten minutes after treatment, cells were analyzed for Fluo-4 fluorescence as an indicator of changes in intracellular calcium (X-axis) and forward light scatter as an indicator of cell size (Y-axis) by flow cytometry. The figure represents a typical experiment.

**Figure 3:**
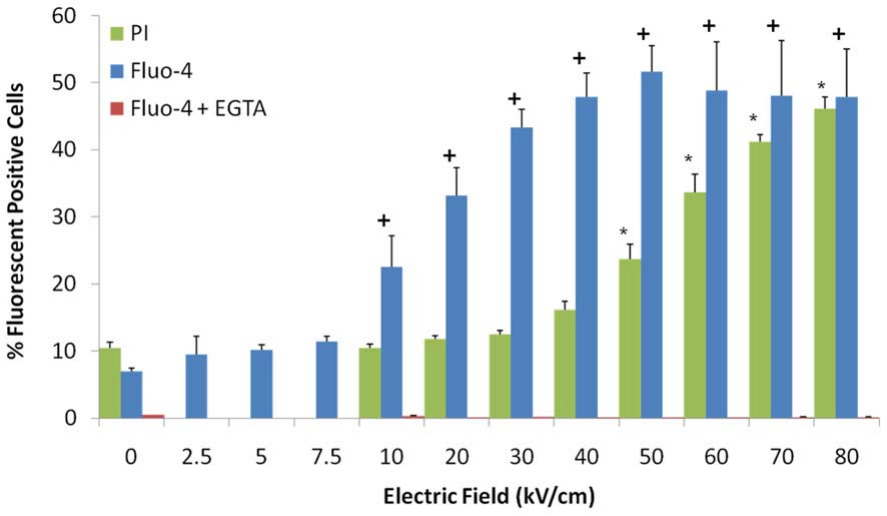
Calcium influx is a better indicator than propidium iodide for nsPEF effects on plasma membranes. Fluo-4 and PI fluorescence were determined in parallel. N1-S1 cells were exposed to single 600 ns pulses at indicated electric fields. For Fluo-4, cells were loaded with the fluorophore as indicated in Experimental Procedures, incubated in the presence or absence of EGTA to chelate extracellular calcium, exposed to nsPEFs and analyzed for green fluorescence by flow cytometry 10 minutes after pulses. For PI, cells were exposed to nsPEFs, PI was added immediately and cells were analyzed for red fluorescence by flow cytometry 10 minutes after pulses. Significant increases in Fluo-4 were observed for all electric fields at and above the 10 kV/cm as indicated by the (+) and for PI above 50 kV/cm as indicated by the (*). There were increases in Fluo-4 florescence in the presence of EGTA. n = 3, p<0.01.

In a typical experiment, Figure 2 analyzes changes in intracellular calcium as indicated by changes is Fluo-4 fluorescence (X-axis) and changes in forward light scatter as an indicator for cell size (Y-axis). Cells were exposed to a single 600 ns short-rise time, square wave pulse without a negative tail. In the absence of EGTA (calcium present; top panels) when nsPEFs are applied, the cell population shifts to the right, indicating increases in calcium at 40 and 80 kV/cm and also shifts slightly up, indicating an increase in forward light scatter, suggesting an increase in cell size or cell swelling. In the presence of EGTA (calcium absent, bottom panels) there is neither a shift to the right nor a shift upward when nsPEFs are applied. This indicates that in the absence of extracellular calcium, increases in intracellular calcium are not detected, signifying an absence of detectable release of calcium from intracellular sources. Figure 3 shows changes in Fluo-4 in the presence and absence of EGTA from several experiments, confirming that there are no measurable increases in intracellular calcium in the presence of EGTA. However, since we and others have shown nsPEF-induced release of intracellular calcium, albeit not in N1-S1 [19]–[21], it is very likely that some level of intracellular calcium is being released, but not detected with Fluo-4.

### Analysis of Plasma Membrane Permeability with Calcium Indicator Fluo-4 Fluorescence: Influx of Calcium Enters Cells through Nanopores at Lower Electric Fields

The presence of nanopores in plasma membranes was previously demonstrated by using thallium, which is much smaller than PI or Yo-Pro [28], which are commonly used to indicate plasma membrane integrity. In Figure 3, fluorescence with the calcium indicator Fluo-4 was compared with fluorescence of PI as measures of plasma membrane integrity. Cells were treated with single 600 ns pulses with 15 ns rise times with increasing electric fields. Fluorescence was determined 10 minutes after treatment with Fluo-4 in the presence or absence of EGTA or with PI in the absence of EGTA (Figure 3). As indicated above, there were no detectable increases in intracellular calcium in the presence of EGTA at any electric field tested. Thus, increases in Fluo-4 fluorescence are primarily due to influx of calcium through plasma membranes. In the absence of EGTA, there were electric field-dependent increases in cells with elevated calcium and PI. Increases in cell numbers with elevated calcium were seen between 7.5 and 10 kV/cm with a plateau at 40–50 kV/cm. Increases in cell numbers with PI were not seen up to values between 40 and 50 kV/cm, reaching a plateau at 80 kV/cm. Given differences in the size of these molecules, influx of calcium becomes a marker for nanopores that are smaller than the dimensions of PI, which are slightly greater than 1 nm [28]. As electric fields are increased, PI fluorescence indicates the appearance of pores larger than a nanometer. Thus, by these determinants, nanopore formation occurs for single 600 ns pulses at electric fields lower than 50 kV/cm. The threshold for nanopore formation here is higher than that previous determined using thallium [28]. While fluorescent microscopy with thallium is more sensitive than fluorescence with Fluo-4 by flow cytometry, the latter approach allows analyses of a large population of cells. As indicated here, only about half of the cells exhibited formation of nanopores. Flow cytometry also provided a measure of the heterogeneity of cell responses to nsPEFs. As indicated in Figure 2, after treatment with nsPEFs, levels of intracellular calcium varied among cells by two orders of magnitude. For those cells exhibiting increases in intracellular calcium, levels varied by an order of magnitude.

### Effects of Different Pulse Transient Features on Mitochondria Membranes and Plasma Membranes in N1-S1 HCC Cells

**Figure 4:**
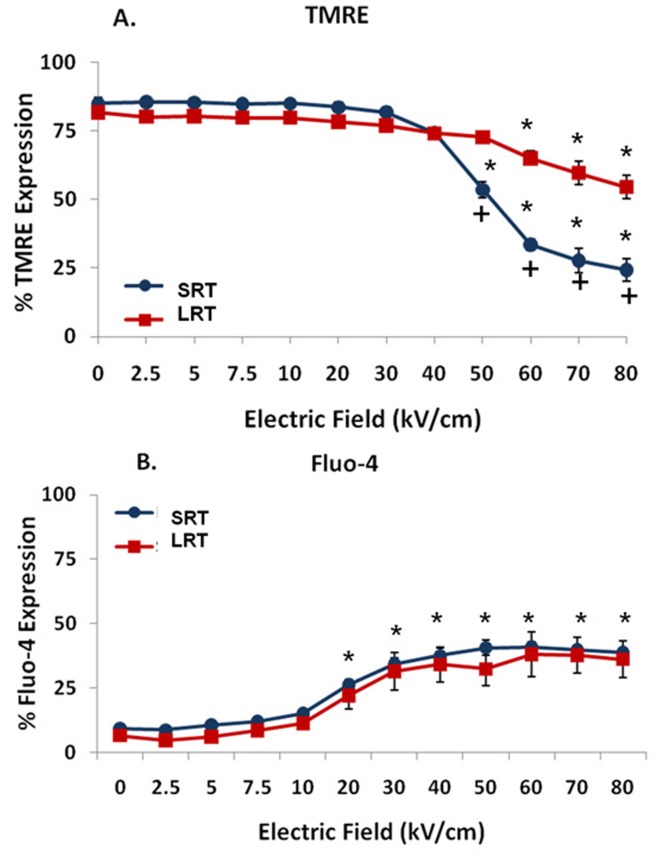
Effect of different pulse shapes on Fluo-4 fluorescence as a marker for plasma membranes and TMRE as a marker for mitochondria membranes. Cells were labeled with the respective markers as indicated in Material and Methods. N1-S1 cells were exposed to single 600 ns pulses at the indicated electric fields with two different waveforms (15 ns rise time and 150 ns rise time) as shown in Figure 1. Effects on ΔΨm with TMRE are shown in panel A; effects on calcium influx with Fluo-4 are shown in panel B. The results represent three separate experiments carried out in duplicate. (#n = 3, p<0.02 vs. 0 kV/cm). Significant differences on both parameters are indicated in Figures 5 and 6.

In order to determine differences between pulse *transient features* on thresholds for nsPEF-induced effects on mitochondria membranes and plasma membranes (Figures 4), cells were exposed to single 600 ns pulses at various electric fields and analyzed for TMRE (Figure 4A) and Fluo-4 fluorescence (Figure 4B). Decreases in fluorescence for TMRE indicate percentages of cells with decreased ΔΨm, while increases in Fluo-4 fluorescence indicate percentages of cells with elevated intracellular calcium levels. The pulse waveform generated by short rise time pulses had the greatest impact on dissipation of ΔΨm (Figure 4A). With electric fields ≥50 kV/cm, significant numbers of cells exhibited dissipated ΔΨm and this was electric field-dependent. Waveforms with long rise times exhibited decreases in ΔΨm, but not as drastically as with short rise times. Differences between pulse waveforms had lesser effects on plasma membranes than those on mitochondria membranes (Figure 4B). Both waveforms had similar increases in calcium influx.

### Effects of Different nsPEF Waveforms on Cell Viability, Plasma Membranes and Mitochondria Membranes in N1-S1 HCC Cells

**Figure 5:**
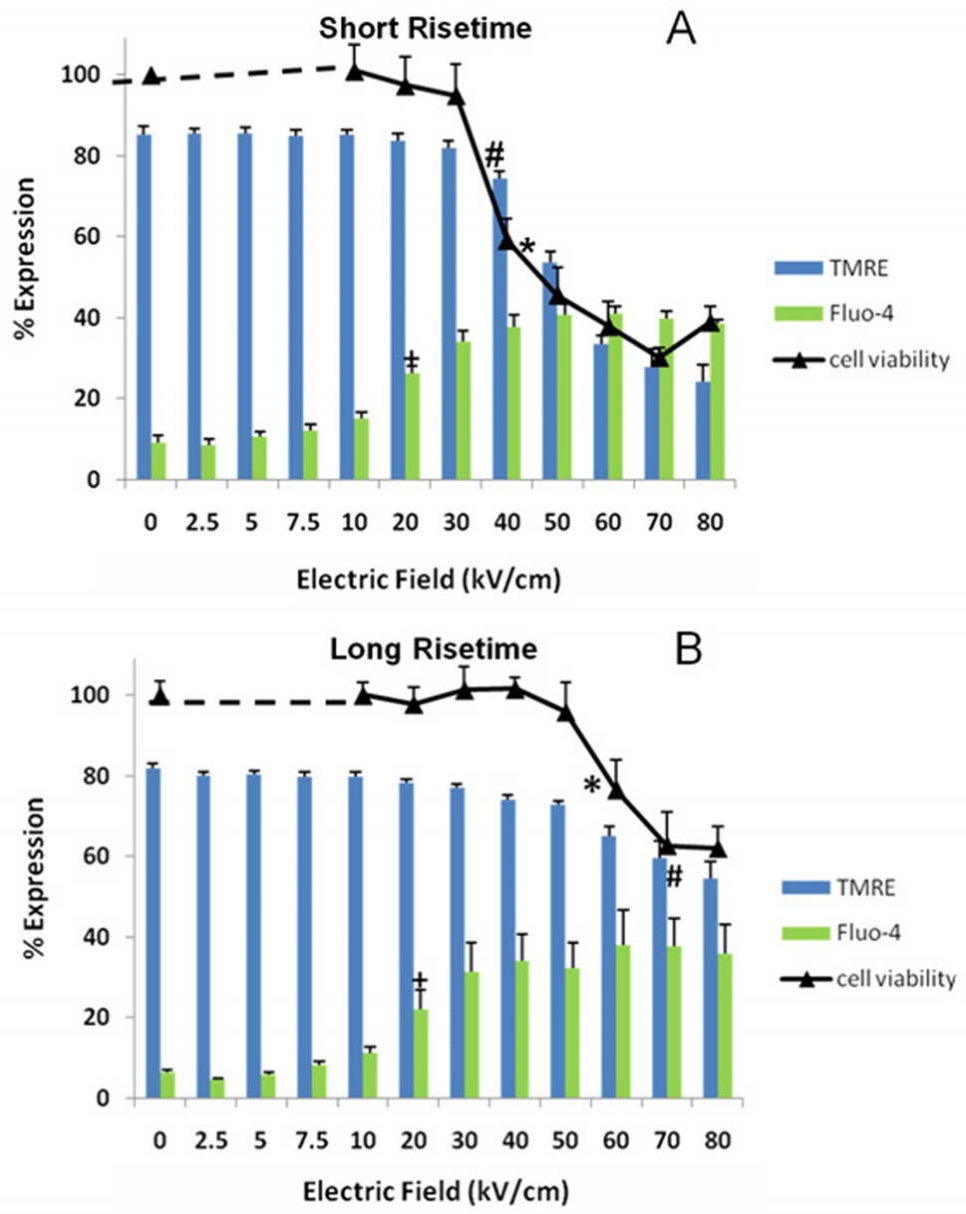
Effects of calcium influx, dissipation of ΔΨm and viability in response to nsPEFs with. Cells were treated and analyzed as indicate in the legend to Figure 4. Effects on increases in calcium influx and loss of ΔΨm were determine 10 minutes after treatment and cell viability was determined 24 hours after treatment (panels A and B) as described in Material and Methods. Panels C and D show corresponding waveforms. Significant differences from control sham treatment are indicated for all electric fields greater than and equal to the symbol for calcium (+n = 3, p<0.001), ΔΨm (*, n = 3, p<0.03) and viability (**#**, n = 3, p<0.001). (Correction: ANOVA with Student-Newman-Keuls).

In order to appreciate the effects of pulse shape on both plasma membrane and mitochondria membrane effects simultaneously on the same image and correlate them with effects on viability, Figures 5 was constructed. As indicated in Figure 4, pulses with short rise times had greatest effects on ΔΨm and here it can be seen to correlate very tightly with cell viability. The threshold for ΔΨm and viability was between 30 and 40 kV/cm. The long rise time pulse had lesser effects on ΔΨm and a corresponding lesser effect on viability. The threshold for ΔΨm and viability was between 50 and 60 kV/cm; this is about 20 kV/cm more than the short rise time pulse. As shown in Figure 4, effects on plasma membrane calcium influx were not significantly different.

### Decreases in ΔΨm are Time-dependent and Enhanced by Calcium

**Figure 6:**
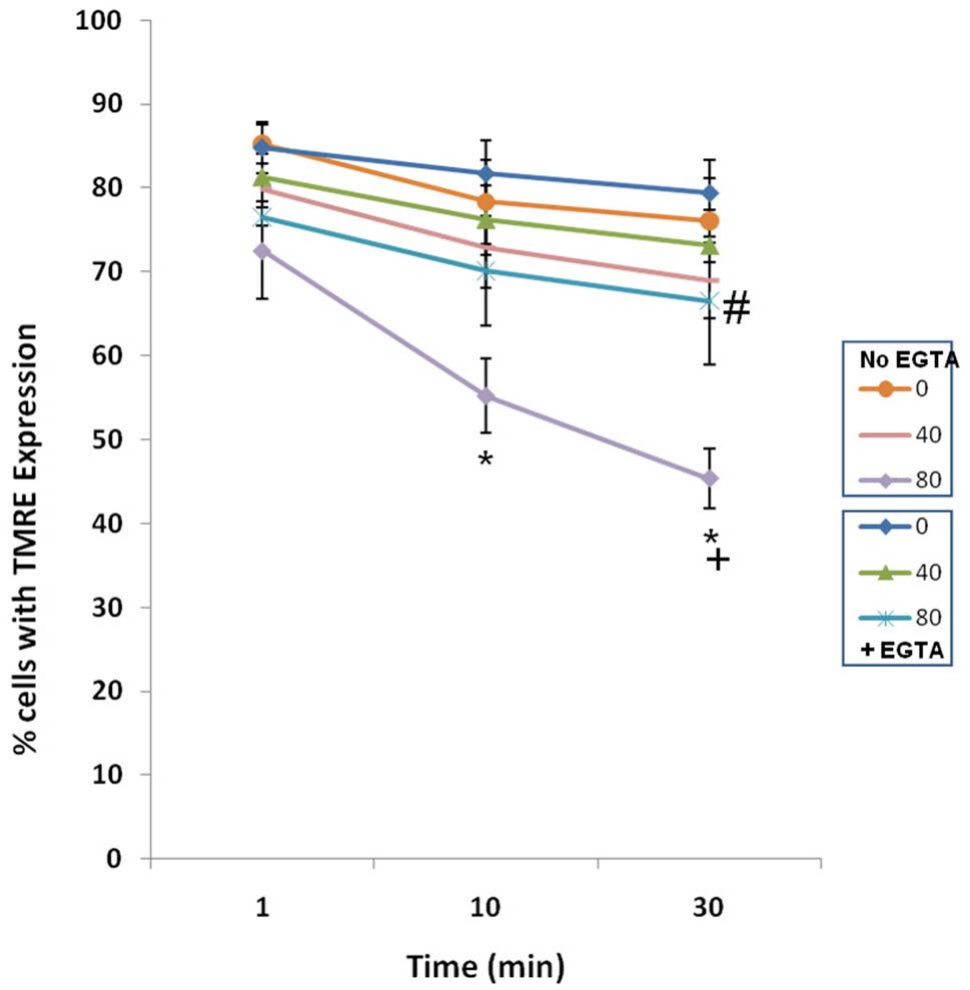
Effects of EGTA and time on ΔΨm. Cells were loaded with TMRE and treated in the presence or absence of EGTA with one 600 ns pulse at the indicated electric fields. Ten and 30 minutes later, TMRE fluorescence was determined by flow cytometry. * = p<0.001 vs. 0 kV/cm at time point; +p = 0.026 vs. 80 kV/cm+EGTA; #p = 0.05 vs 0 kV/cm +EGTA.

In Figures 2, 3, 4, 5, ΔΨm was determined 10 minutes after nsPEF treatment. Figure 6 shows 1, 10 and 30 minute time points for changes in ΔΨm with increases in electric field in the presence and absence of EGTA to chelate extracellular calcium. The short-rise time pulse waveform was used; single 600 ns pulses were applied at 40 or 80 kV/cm. In the presence of calcium (- EGTA), there were electric field-dependent and time-dependent dissipations of ΔΨm with greater effects at 80 kV/cm. However, in the absence of calcium (+EGTA), there was the only a statistically significant decrease in ΔΨm 30 minutes after nsPEF treatment. These data suggest that the primary nsPEF-induced dissipation of ΔΨm is time-dependent and calcium-dependent and that there is a calcium-independent effect that can be seen between 10–30 minutes after nsPEF treatment.

## Discussion

One of the major findings in this report is the striking differences nsPEF fast rise and fall waveforms have on mitochondria membrane potentials, ΔΨm. This study makes clear that pulses with short rise and fall times and 600 ns in duration are most effective for modifying intracellular membranes as shown here using ΔΨm as a mitochondria membrane marker. Since shortening rise and fall times correspond to a shift to higher frequencies in the Fourier spectrum of nsPEFs, the data provide the most direct evidence for confirmation of the hypothesis that high frequency components impact intracellular membranes.

In contrast to effects on ΔΨm, transient features or high frequency components had much less effects on plasma membranes. Both pulse waveforms regardless of rise and fall times exhibited similar electric field-dependent effects on plasma membranes. It is also significant to note that regardless of rise time, permeabilization of plasma membranes occurred at lower electric fields than dissipation of ΔΨm, indicating that as electric fields are increased, plasma membranes are more sensitive responders than mitochondria membranes.

The second major finding in this study is the close correlation between dissipation of ΔΨm and loss of cell viability. As pointed out above for ΔΨm, the fast rise and fall waveforms had a dramatic effect on viability, while the slow rise and fall waveforms had lesser effects on viability and on ΔΨm. This indicates that nsPEFs target mitochondria membranes with dissipation of ΔΨm as a major factor initiating cell death. This is in contrast to studies using gadolinium concluding that plasma membrane permeability is a likely mechanism of lethal cell damage [29]. However, another important point to note is that intracellular calcium, coming primarily through plasma membranes in N1-S1 cells, significantly potentiates dissipation of ΔΨm and thereby potentiates cell death. NsPEF-induced calcium release from ER has been shown before in other cells [19], [21], but was not evident here. A possible reason for these results may be release of calcium in micro-domains with concentrations high enough to activate calcium uniporters in inner mitochondria membranes [30], [31], but below the sensitivity of Fluo-4. Nevertheless, there are some well-characterized proteins that participate in tight mitochondria-ER interfaces and calcium communications for cell death regulation between these two organelles have recently becoming clearer [32]. It would be of interest to know if ER and mitochondria responded with similar thresholds or were affected similarly by high frequency components or if they could be differentially regulated by these factors.

Increases in intracellular calcium and dissipation of ΔΨm with fast rise and fall waveforms could be explained by direct electric field effects to nanoporate plasma membranes and inner mitochondria membranes. Within the first minute after nsPEF application (the shortest time measurable here), the presence or absence of calcium makes little difference in the fall of ΔΨm; however, these decreases are relatively small and decreases in ΔΨm in the absence of calcium is only significant 30 minutes after treatment, which is not typical of a direct effect; nanoporation of the inner mitochondria membrane would be immediate and likely calcium-independent. In contrast, the greatest effect on ΔΨm is significantly calcium-dependent and also time-dependent. This suggests that nsPEFs may affect the mitochondria membrane permeability transition pore (mPTP) complex [35], which is both calcium- and electric field-dependent. This suggests mechanism(s) that are independent of inner mitochondria membrane poration, but dependent on increases in intracellular calcium, most likely after plasma membrane poration. It is then likely that elevated intracellular calcium causes time-dependent increases in calcium uptake by mitochondria resulting in calcium overload, eventual opening of the mPTP and collapse of ΔΨm [36]. Regardless of what causes these effects on ΔΨm, cell swelling could ultimately cause mitochondria membranes to rupture and cytochrome c release and dissipating ΔΨm would cause ATP levels to decrease; cells would die by apoptosis, necroptosis and/or necrosis, depending on calcium levels [33] and/or ATP levels [34]. Interestingly, increases in forward light scatter as an indicator of cell swelling did not occur in absences of calcium, suggesting that cell swelling in response to nsPEFs may also be enhanced by calcium.

These studies using flow cytometry and calcium ions as indicators for plasma membrane effects with single 600 ns pulses are in agreement with studies reported previously using a Tl(+)–sensitive fluorophore and microscopy to analyze plasma membrane effects [28]. While it is difficult to determine the real size of calcium as a “nanomolecule” because of hydration layers, both studies clearly demonstrate that pores formed in plasma membranes can be considerably less than a nanometer, at least at electric fields <50 kV/cm in this study. In addition, because cell types, buffer systems, cell densities and analytic tools were different, this study confirms formation of nanopores in plasma membranes. Microscopic examination of plasma membrane provides a greater sensitivity than flow cytometric analysis as indicate by thresholds for nanopore formation of 1–2 kV/cm for Tl(+) [28] vs. 7.5–10 kV/cm for calcium observed here. Microscopy also allows sharply defined kinetic analyses of transport across membranes that are limited by flow cytometry. The studies here monitored nanopore formation with calcium as a physiological regulator and second messenger and observed large cell populations. Interestingly, only about half of cells responded with increases in calcium or PI, even with electric fields as high as 80 kV/cm, which is about as high as can be achieved before electrical breakdown occurs.

These studies present new perspectives for transient features of nsPEFs or their high frequency components on cell membranes, and expand initial considerations for rise and fall time on cell membranes [1] to include effects on cell viability. The results make clear that high frequency components of nsPEFs can differentially modulate intracellular structures as revealed by inducing dissipation of ΔΨm and thereby selectively influencing cell viability. Given that nanoporation of plasma membranes occurs at lower electric fields than dissipation of ΔΨm, calcium is always present when effects on ΔΨm occur. Thus, nsPEFs affect plasma membrane poration and calcium influx as well as affect calcium-dependent mechanisms in mitochondria, likely through the mPTP complex.

It is a common interest of whether or not nsPEFs can selectively affect cancer cells and spare normal cells. While there is some selectivity for nsPEFs on different cell types, it remains to be shown that such differences extend to normal versus cancer cells. When electric fields are high enough, as they need to be during ablation therapy, all cells in electric fields that are above a threshold for effects on ΔΨm will die by more than on mechanism [7], [8]. Confining the treatment only to cancer cells may be obtained by placing the electrodes in the cancerous tissue and in the meantime creating a large electric field gradient from cancerous tissue to normal tissue using needle electrodes. As a result, a relatively well-defined ablation zone may be expected [37]. The correlation of electric field intensity and cell death become a priori in order to define the effective treatment zone and spare normal tissue.
